# A two-tiered methodology for the validation of promising plant growth promoting bacteria isolated from durum wheat rhizosphere

**DOI:** 10.3389/fpls.2025.1707549

**Published:** 2025-11-06

**Authors:** Annalisa d’Amelio, Michele Andrea De Santis, Luigia Giuzio, Damiana Tozzi, Daniela Campaniello, Angela Racioppo, Maria Rosaria Corbo, Zina Flagella, Antonio Bevilacqua

**Affiliations:** Department of Agriculture, Food, Natural Resources and Engineering (DAFNE), University of Foggia, Foggia, Italy

**Keywords:** biofertilizer, selection, from lab to field, persistence, promoting effects

## Abstract

**Introduction:**

Plant growth promoting bacteria (PGPB) are gaining increasing attention as a sustainable tool to support crop performance under environmental and agronomic stress conditions. However, the transition from laboratory to field is a challenge.

**Methods:**

Six PGPB strains from the rhizosphere of durum wheat were evaluated for their technological characteristics and agronomic performance, to identify suitable candidates for field conditions in a Mediterranean environment. The strains were studied for their resistance to two commercial fungicides, ability to persist in soil at different temperatures, and their growth over pH and temperature.

**Results and discussion:**

The strain 23P (*Pseudomonas migulae*) showed the best overall performance, with the highest resistance to fungicides and viability in soil; in addition, this microorganism enhanced shoot biomass and nitrogen uptake, increasing shoot-to-root ratio and N:P. The results support a selection strategy that includes both technological characterization and agronomic validation with a comprehensive and holistic approach from lab to field.

## 1 Introduction

According to the Food and Agriculture Organization of the United Nations (FAO), global agricultural production must increase by 60% by 2050 to meet the rising food demand driven by population growth (Cao et al., 2023; Fadiji et al., 2022). Of this increase, 90% is expected to come from agricultural intensification, while only 10% will result from the expansion of arable land, presenting a challenge for the sustainability of primary production (Di Benedetto et al., 2017).

The aim of modern agriculture is, therefore, to find sustainable solutions, especially in the Mediterranean regions where weather conditions play a major role (De Santis et al., 2024).

Plant Growth Promoting Bacteria (PGPB) represent one of the most promising strategies to achieve this goal (Racioppo et al., 2023; Shah et al., 2021). These microorganisms colonize the rhizosphere and interact with host plants as microbial symbionts, feeding on root exudates. Although the mechanisms by which PGPB promote plant growth are known, a comprehensive understanding of the mechanisms by which they adapt to environmental conditions and colonize soil is lacking (Wang et al., 2023). During application, PGPB could also encounter stress or harmful conditions, due to the way of applications, the persistence in soil, or the use of fungicides or other compounds, which could also play an antibacterial activity. Moreover, it is recommended that they could act also under challenging conditions, for example in the case of hot stress, which is now a common condition in Southern Italy (Carillo, 2025). In fact, it is demanding to verify that the improvement to plant growth might occur not only under controlled experimental conditions but may also lead to agronomic benefits for crops cultivated under open field conditions (Caldara et al., 2024); one of the barriers for an effective application of PGPB is the scarcity of field-level knowledge (Gopalakrishnan et al., 2015; Hossain et al., 2023). The selection of PGPB starts in laboratory and the optimization of formulations relies upon pot-experiments, but laboratory experiments need to be transferred from pot experiments to field-level trials (Hossain et al., 2023), and to best of authors’ knowledge, only a few studies propose a structured protocol from laboratory to farm, with a comprehensive focus on growth, resilience, easiness of use, performances in a small-scale and in field.

Cereal crops represent the staple basis for human nutrition; durum wheat represents a candidate target crop, due to its relevance in Mediterranean cropping systems (Grosse-Heilmann et al., 2024), and a promising activity on the use of PGPB as plant biostimulant on durum wheat is reported (Grosse-Heilmann et al., 2024), in relation to environmental stresses and mineral fertilization (Rossini et al., 2025; Spada et al., 2024), thus suggesting this approach as a promising method for this crop.

A preliminary activity of selection of promising PGPB strains was carried out by our group (Di Benedetto et al., 2019), especially in relation to nitrogen and phosphorus use efficiency; as a result, 16 different strains, belonging to *Bacillus*, *Pseudomonas*, *Stenotrophomonas*, and *Lysinibacillus* genera were isolated, characterized for some promising PGPB traits (siderophore and ammonium production, phosphate solubilization, phosphate mineralization, production of indole acetic acid, nitrification), preliminary tested in a growth chamber (Di Benedetto et al., 2019), and some of them on durum wheat under phosphate starvation conditions (Cataldi et al., 2020). However, a further characterization of the adaptability of these wild strains and their efficacy on agronomic conditions is required, along with an assessment of their suitability in harsh conditions, as well as their resistance and easiness of use in real conditions.

Therefore, this paper proposes the characterization and optimization of promising PGPB, through a two-tiered methodology. First, a protocol to assess the technological robustness is proposed, focusing on soil persistence also under increasing temperature conditions, and resistance to fungicides, along with the evaluation of growth profile as a function of pH and temperature. Then, a sequential approach based on pot and field trials is outlined for strain validation and to assess their effectiveness under controlled and field conditions, aiming at improving nutrient use efficiency, and thereby exerting a beneficial effect.

## 2 Materials and methods

### 2.1 Microorganisms

Six strains from the rhizosphere of durum wheat, as described by Di Benedetto et al. (2019), were used in this study (**Table 1**). All the strains were stored for short term maintenance at 4°C on Nutrient Agar (Oxoid, Milan, Italy), while long term storage was done at -80°C in Nutrient broth, supplemented with 33% sterile glycerol (J.T.Baker, Milan); the microorganisms were cultured prior to each assay under aerobic conditions in Nutrient Broth at 25-30°C, depending on the microorganism, for 24–48 h.

**Table 1:** Test strains.

Identification number	Genus/species	Accession number
12A	*Bacillus* spp.	MG515472
20P	*Stenotrophomonas* spp.	MG515465
23P	*Pseudomonas migulae*	MG515462
25A	*Bacillus* spp.	MG515463
36M	*Bacillus* spp.	MG515459
40M	*Bacillus* spp.	MG515460

### 2.2 Resistance to commercial fungicides

Bacterial suspensions were prepared in sterile isotonic solution (0.9% NaCl) at 7 log CFU/mL, and supplemented with different concentrations (1 to 4 g/L) of two commercial fungicides, Vibrance Gold (Difénoconazole 25 g/L; Fludioxonil 25 g/L; Sedaxane 50 g/L; Syngenta Italy Spa, Milano, Italy) and Celest Trio (Difénoconazole 25 g/L; Fludioxonil 25 g/L; Tebuconazole 10 g/L; Syngenta Italy Spa, Milano, Italy). Inoculated saline solutions but without commercial fungicides were used as controls. All samples were incubated at the optimum temperature for each strain and analyzed after 24 and 48 hours to assess viable count on Nutrient Agar.

### 2.3 Persistence in soil

Thermally-treated soil samples (200 g) (Terra Pastorizzata; Italiana Terricci, Merate LC, Italy) were inoculated at 7 log CFU/g (10 mL of bacterial suspension for each sample) and placed in sterile containers, covered with sterile cotton wood; soil characteristics were the following, as reported by the producer: pH 7.80; EC, 1.60 dS/m; P, 20 mg/g of dry weight; organic matter, 20 g/kg of dry weight; clay, 175 g/kg dry weight. The samples were incubated at 5, 15, 25, 35 and 45°C in incubators equipped with vent aerators and viable counts on Nutrient Agar assessed after 15, 30, 45 and 60 days. Data were modelled as viability loss.

### 2.4 Effect of pH and temperature on growth profiles

Bacteria were inoculated at 7 log CFU/mL into Nutrient Broth at different pHs (from 4.5 to 9) and incubated at 25°C and 30°C for 24 and 48 hours. Additionally, bacteria were inoculated into standard Nutrient Broth and incubated at 15, 25, 30 and 35°C. Bacterial growth was evaluated through absorbance measurement at 600 nm; data were then modelled as *Growth Index* (GI), modified by Bevilacqua et al. (2009).

### 2.5 Seed attachment assay

*Triticum durum* seeds were surface sterilized as reported by Di Benedetto et al. (2019). A seed was then placed in contact with bacterial suspension (1 mL, 7 log CFU/mL) for 60 min, then removed, air dried for 30 min and transferred to microtubes containing 1 mL of sterile distilled water. The tubes were vortexed for 2 min to promote detachment of cells. Serial decimal dilutions of the washing water were made and plated on nutrient agar. Data were processed as adhesion efficiency (E%) (concentration of the microorganism in the washing water *vs* bacterial suspension).

### 2.6 Growth chamber

Bacterial suspensions were preliminary standardized to 7 log CFU/mL through OD_600nm_ measurement. Durum wheat seeds (both Marco Aurelio and Saragolla) were treated at a rate of 1 mL for seed for 60 min; seeds treated with sterile buffer represented the control. Then bacterial suspensions were also used for direct soil inoculation, close to the seed (1 mL for seed).

A randomized block design with 4 replications was used; the design consisted of two durum wheat genotypes (Marco Aurelio and Saragolla) and seven treatments (PGPB+control). Ten seeds were sown per pot (3.6 L). The sowing density corresponded to 240 seeds/m^2^ (6 seeds per pot) while harvest was done after 76 days at the beginning of heading stage (BBCH 51). Detailed growing conditions are reported in **Table 2**. Crop development was described in terms of days after sowing (DAS), phenology (BBCH) and growing degree days (GDD). Plant height (PH) was measured at different DAS (19, 27, 40, 58 and 76). At heading (76 DAS) shoot and root dry matter biomass were determined by collecting all plants and expressed as mg/m^2^, determined on a pot surface area of 0.0225 m^2^; shoot to root ratio was also calculated. Nitrogen (N, CHNS elemental analyzer) and phosphorus (P, ICP-OES) concentration were determined in shoot tissues, then N and P uptake were determined by multiplying N and P concentration to SB. The ratio between N and P uptake (N:P) was also determined.

**Table 2:** Details of trial in growth chamber.

DAS	Growth stage	BBCH	T max	T min	GDD	Water supply
d	GS		°C	°C	°C d	L/m^2^
0	sowing	00	12	12	12	0
8	start emergence	02	16	12	110	11
14	emergence	05	12	8	182	11
19		11	12	8	232	17
27	2 leaves	12	12	8	312	27
34	3 leaves	13	12	8	382	32
40			12	8	442	34
43	tillering	21	15	10	475	36
53			15	10	600	38
57	stem elongation	31	15	10	650	41
58			15	10	662	41
69	booting	45	18	12	822	50
76	early heading	51	18	12	927	54

DAS, days after sowing; GS, growth stage; BBCH, decimal phenological code; T max, maximum temperature; T min, minimum temperature; GDD, growing degree days.

Soils samples were collected at sowing and booting; total bacterial count, aerobic and anaerobic spore forming bacteria, nitrogen fixing bacteria, pseudomonads, actinobacteria and soil bacteria grown at 22°C were analyzed as reported by De Santis et al. (2023). The pH was also determined on the samples.

### 2.7 Field trial

23P strain was evaluated in a field trial. The experiment was conducted in two close fields (2.8 km) in the province of Foggia (Italy), respectively at Bovino (S1, 41.289667 N, 15.444639 E) and Castelluccio dei Sauri (S2, 41.270971 N, 15.465364 E). Details of soils characteristics are reported in **Table 3** while weather conditions are reported in **Table 4**. A randomized block design with three replications with two durum genotypes (Marco Aurelio and Saragolla) and two PGPB treatments (23P *vs* control) was adopted, with a plot size of 4.5 m^2^ (1.5 x 3.0 m). Sowing was carried out at a rate of 450 seeds per m^2^ and mineral fertilization consisted in the application of 140 kg/ha of nitrogen and 50 kg/ha of phosphorus, as detailed in De Santis et al. (2024). Herbicides (mesosulfuron methyl 15 g/ha, iodosulfuron-methyl-sodium 3 g/ha) and fungicides (azoxystrobin, 23 g/ha) were applied at stem elongation. At tillering, spray inoculation of the 23P strain (10^7^ CFU/mL) was carried out in the treated plots. At maturity (194 days after sowing) plants were randomly collected for the determination of plant height (PH) and harvest index (HI); then, grains were harvested by plot combine and grain yield (GY) was determined. Nitrogen (N, CHNS elemental analyzer) and phosphorus (P, ICP-OES) concentration were determined in grains, then N and P uptake were determined by multiplying N and P concentration to GY. The ratio between N and P uptake (N:P) was also determined. Further, N and P use efficiency (NUE and PUE, respectively) were assessed in terms of output/input ratio, i.e. as the ratio between mineral uptake on the fertilization rate. Microbiological analyses were done as previously reported.

**Table 3:** Information on soil characteristics for field experiments.

Soil	Fertility	Depth	Sand	Silt	Clay	pH	Organic matter	Total N	Available P
		m	%	%	%		%	g/kg	ppm
S1	low	0.6	26.4	52.8	15.1	8.1	3.02	0.66	43.1
S2	average	< 1.0	36.8	8.8	54.4	7.9	2.92	0.75	29.3

**Table 4:** Monthly weather for field trials: S1, Bovino; S2 Castelluccio dei Sauri. Data includes monthly cumulative growing degree days (GDD) and precipitation (P).

Month	T max	T min	GDD	P
	°C	°C	°C d	mm
January	11.8	2.0	207	39
February	13.9	3.5	244	107
March	13.0	1.8	229	47
April	17.9	6.1	360	14
May	26.7	12.4	608	32
June	33.2	17.7	765	58

### 2.8 Statistical analysis

All tests were performed at least twice on two independent samples, for each combination and for each strain. Multifactorial Analysis of Variance (MANOVA) and Tukey’s *post-hoc* (p<0.05) were used for statistical analysis of the results; the effect of PGPB strains on wheat growth in controlled conditions (growth chamber) was evaluated by hierarchical cluster analysis (Ward method). Statistic was done using the software Statistica for Windows ver. 12.0 (Statsoft, Tulsa, Okhla).

## 3 Results

### 3.1 Fungicides

**Figure 1:**
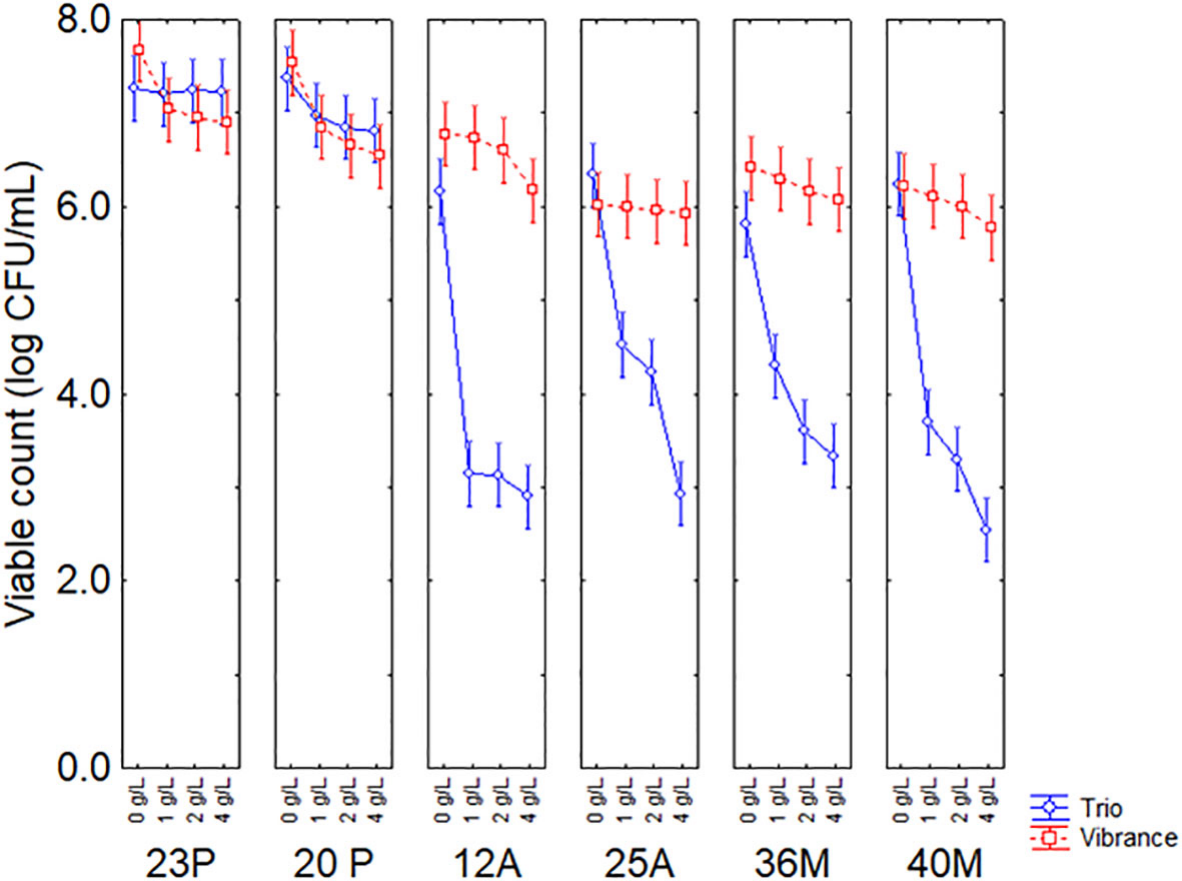
Interaction concentration *vs* fungicide *vs* microorganism on the viable count of the target strain after 48 h. Bars represent 95% confidence intervals.

All predictors were significant, with a higher statistical weight attributed to the type of fungicide (F-test, 620.83), followed by the strain (F-test, 251.39) and finally by the fungicide concentration (F-test, 134.41). However, the table of standardized effects only provides qualitative results, whereas quantitative results could be obtained by the figures on the decomposition of the statistical hypothesis. **Figure 1** shows the decomposition of statistical hypotheses for the interactive term concentration *vs* fungicide *vs* microorganism on the viable count of the target strain after 48 h of contact time with the commercial fungicides at concentrations from 0 to 4 g/L. Strains 23P and 20P were the most resistant microorganisms to both fungicides with an average concentration of 6–7 log CFU/mL. In contrast, strains 12A, 25A, 36M and 40M showed a drastic viability loss (3–4 log CFU/mL), especially in the presence of Celest Trio. The significant bioactivity of Celest Trio compared to Vibrance was observed even at the lowest concentration tested in this research (1 g/L).

### 3.2 Soil persistence

**Figure 2:**
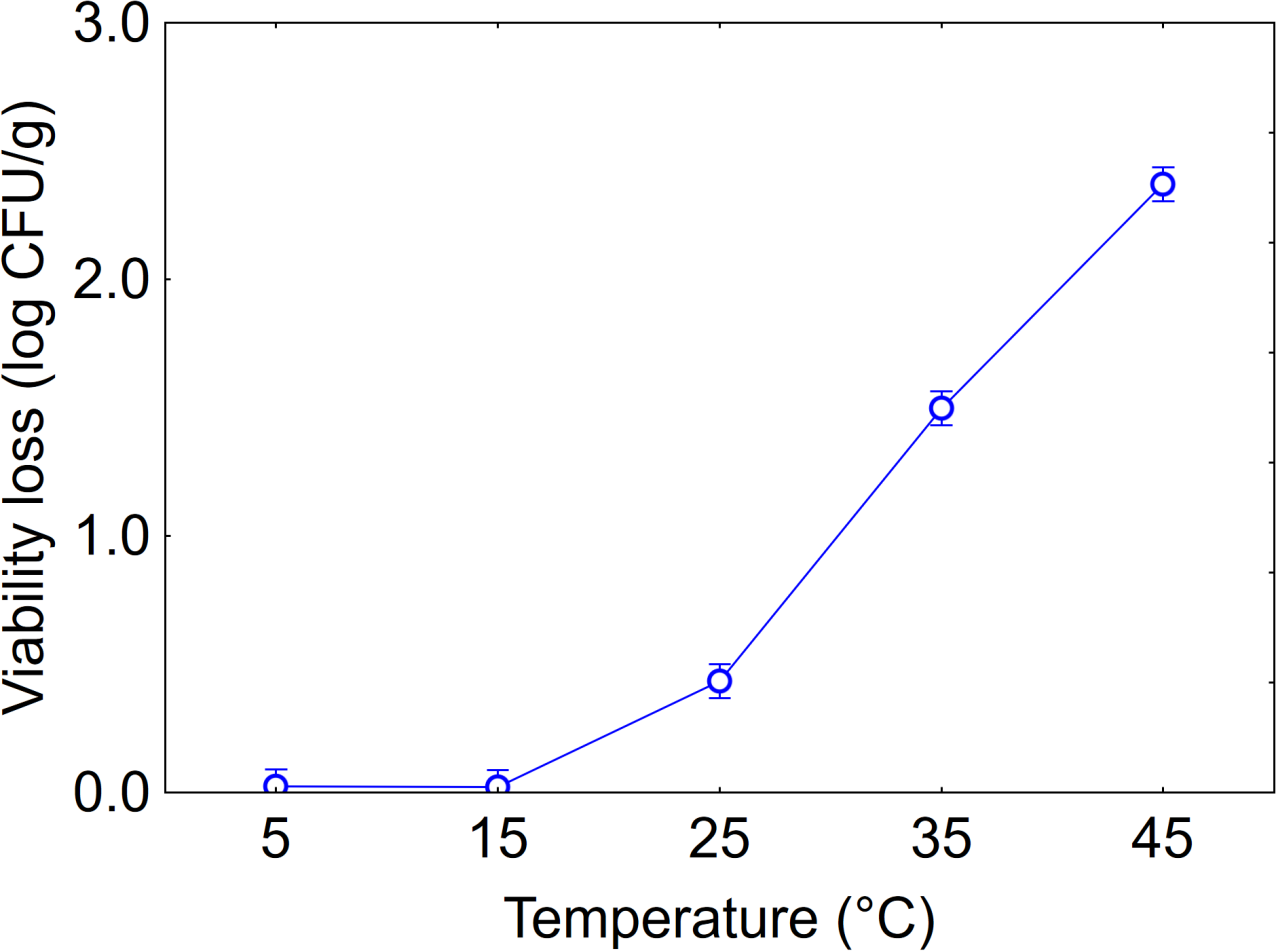
Effect of temperature on the viability loss in soil. Bars denote 95% confidence intervals.

Statistical analysis revealed the significance of the predictors both as individual variables and in their interactions with temperature having the highest statistical weight (F-test, 1282.82), followed by time (F-test, 131.89) and strain (F-test, 63.98). The effect of temperature is shown in **Figure 2**. At low temperatures (5, 15 and 25°C), viability loss was minimal (max. 0.3 log CFU/g), whereas increasing the temperature to 35°C and especially to 45°C caused a significant decline, with average values of 1.6 and 2.4 log CFU/g respectively.

**Figure 3:**
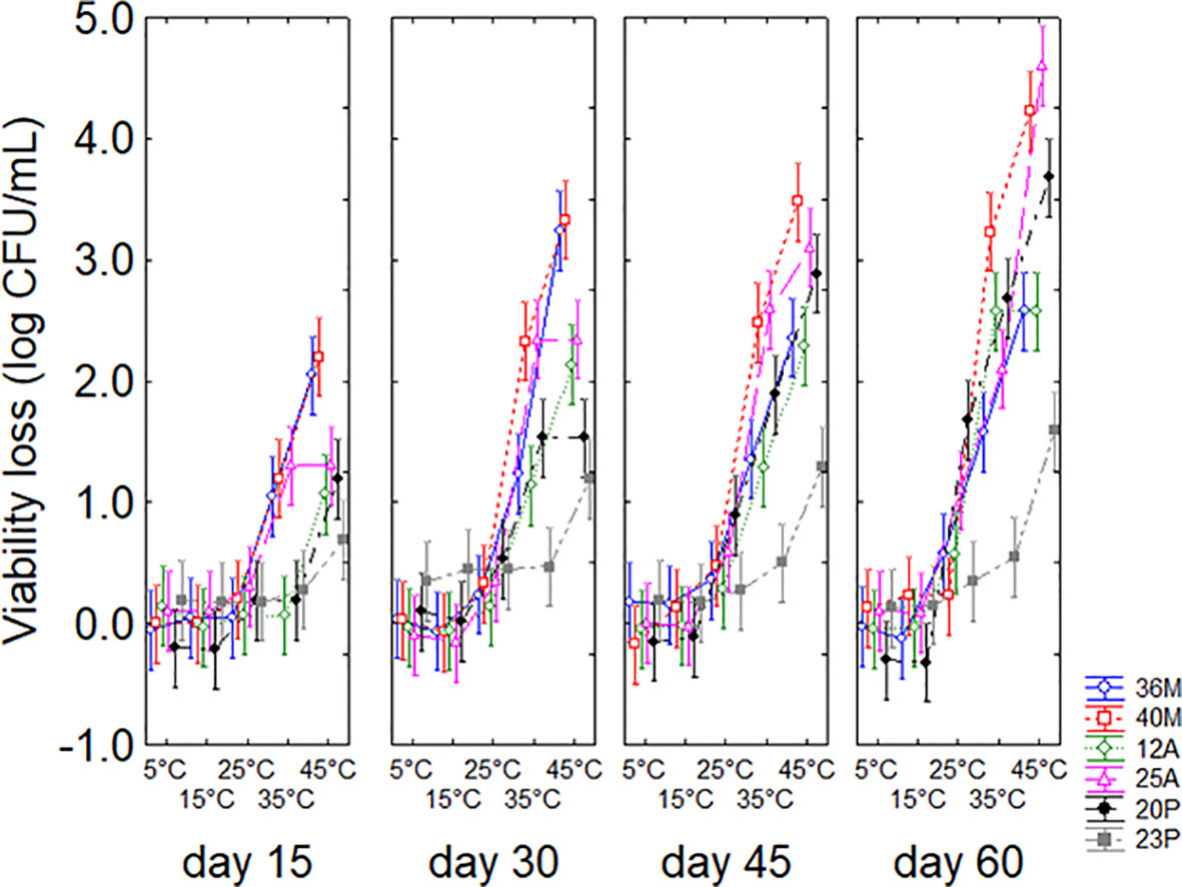
Interaction temperature *vs* microorganism *vs* time on the viability loss in soil. Bars denote 95% confidence intervals.

Statistical hypotheses for the interaction (**Figure 3**) confirmed that both time and temperature play a decisive role. Time contributed, with an increasing loss of viability during the experiment, up to a predicted maximum of 4.5 log CFU/g after 60 days. However, an interesting finding was pointed out for strain 23P, which showed a significantly lower viability loss than the other strains both at 35°C (0.6 log CFU/g) and 45°C (1.3 log CFU/g), indicating a remarkable persistence capacity of this microorganism over time.

### 3.3 Growth profile

**Figure 4:**
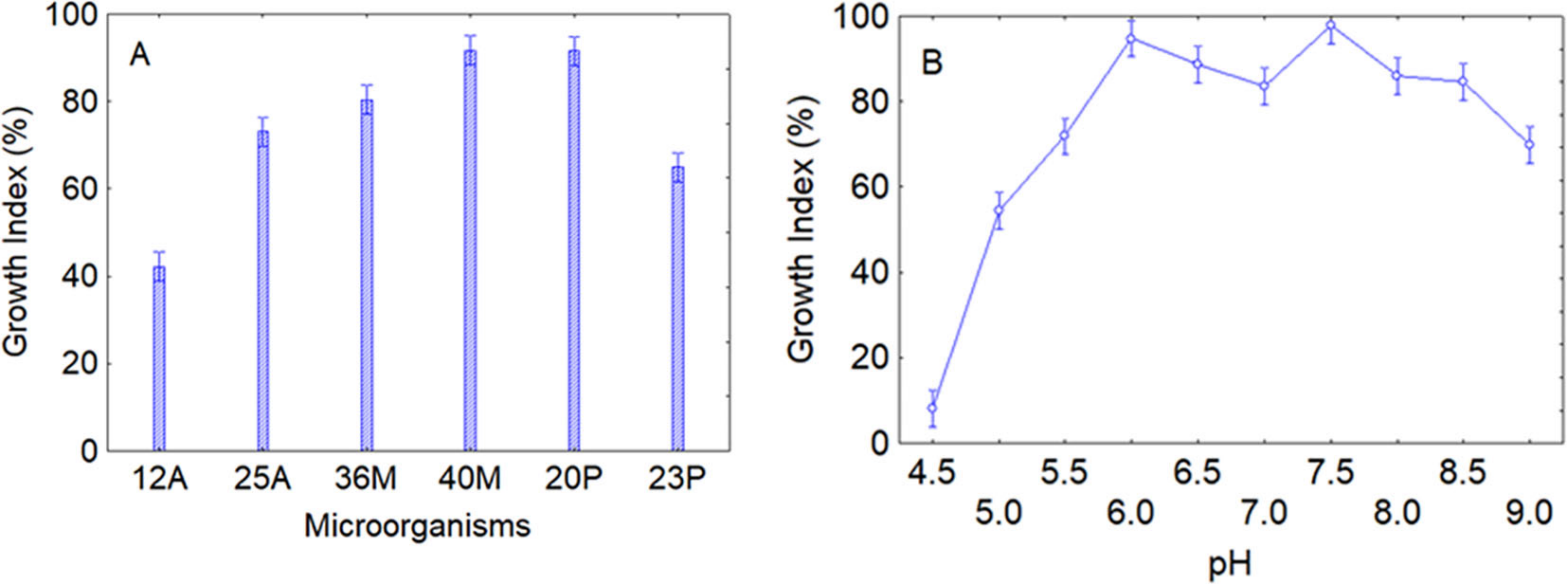
Effects of microorganisms **(A)** and pH **(B)** on the growth index after 48 (h) Bars denote 95% confidence intervals.

After assessing the resistance to fungicides and persistence in soil, the robustness of the strains was evaluated as growth profile as a function of pH and temperature in a laboratory medium; the use of controlled conditions was a choice to avoid possible confounding effects. The first test was on pH; data analysis through the decomposition of the statistical hypothesis clearly identified that growth profile at the different pHs was affected by both the intrinsic resistance of the strains and the effect of pH itself. Concerning the intrinsic resistance of microorganisms, an average increase in microbial concentration (GI>50%) was observed after 48 h of incubation, thus suggesting that the strains could generally grow under various pH levels, but the highest increase was observed for the strains 40M and 20P (GI>95%) (**Figure 4A**). As reported above, the pH itself was the second predictor affecting microbial growth; as expected, the effect of this predictor was drastic at pH 4.5 (GI<20%) for all strains, while the optimum pH values were around neutral values. However, at pH 9 there was again a downward trend in the curve (GI<70%) (**Figure 4B**).

**Figure 5:**
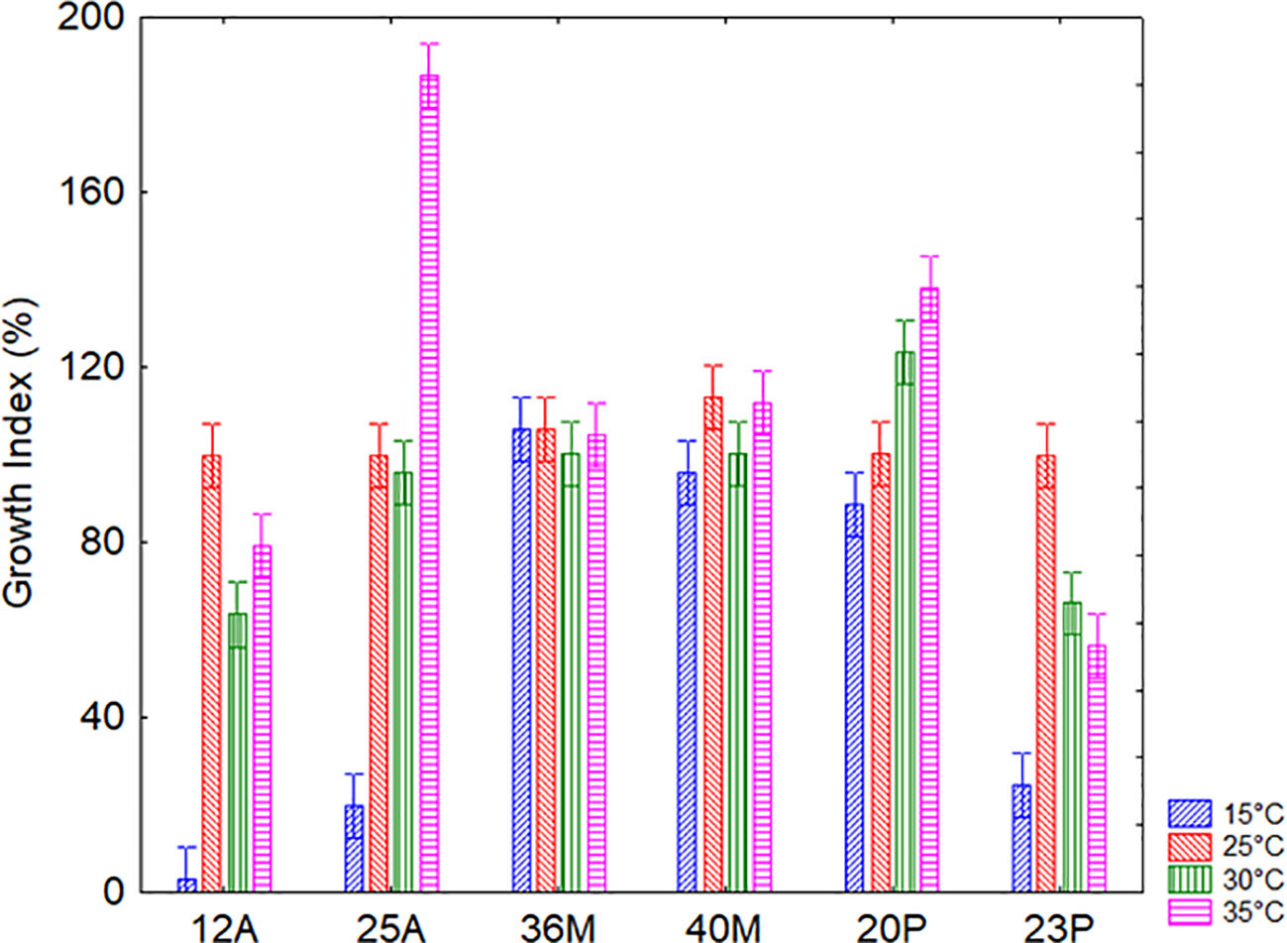
Interaction temperature *vs* microorganisms on the *Growth Index* of the target strains after 24 h for the assay on temperature. Bars denote 95% confidence intervals.

The second factor assessed for technological robustness was temperature. This experiment was different from the results reported in the previous section on soil persistence, as they focus on the ability to grow at different temperature levels under optimal conditions, while section 3.2 reports data for temperature resilience in soil. A significant correlation between temperature and microorganism on the growth profile was detected (**Figure 5**), and the microorganisms responded differently. Microbial growth was less favored at 15°C; however, the trend was significantly strain dependent.

It is important to note that all strains were able to grow in a wide variety of conditions; GI, in fact, is a standardized measure which reports growth data as a function of control, but values >25% indicate growth. Only the strain 12A could not grow at 15 °C (mean GI of 3%) at least for the duration of the experiment.

### 3.4 Adhesion to seeds

**Figure 6:**
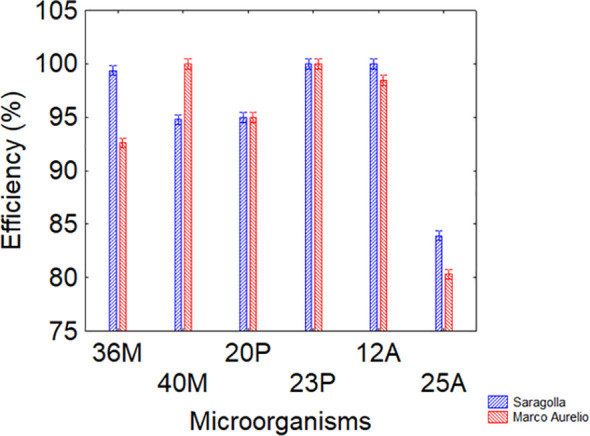
interaction variety *vs* microorganisms of adhesion to seeds. Bars denote 95% confidence interval.

The results indicate that, regardless of the variety (Saragolla or Marco Aurelio), all strains exhibited adhesion efficiencies ranging from 94 to 98%, except for strain 25A, which showed a lower efficiency (80-85%). These findings confirm the possibility of using seed conditioning for an effective use of PGPB (**Figure 6**).

### 3.5 Impact of PGPB inoculation on durum wheat

**Figure 7:**
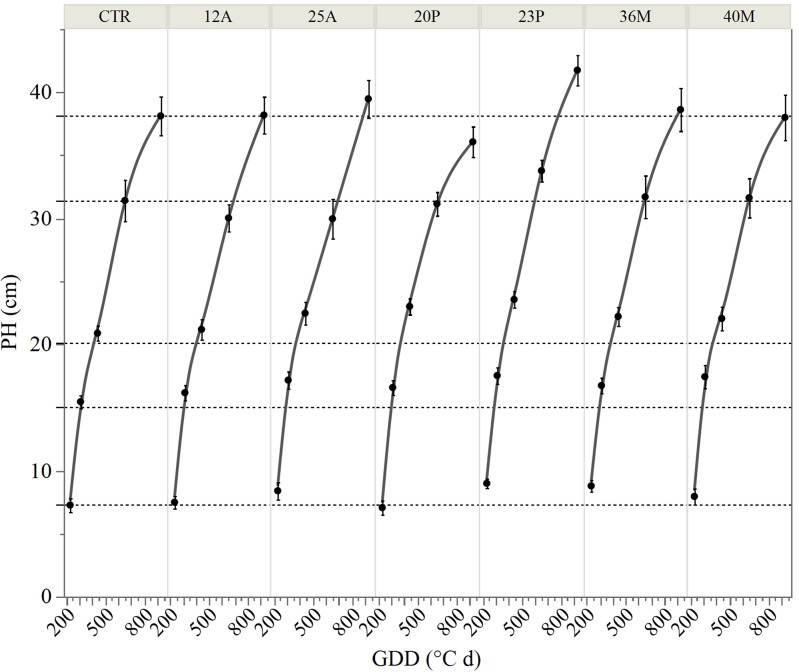
Trend of plant height (PH) at different days after sowing and different growing degree days (GDD). CTR, control; bars represent 95% confidence intervals.

**Figure 8:**
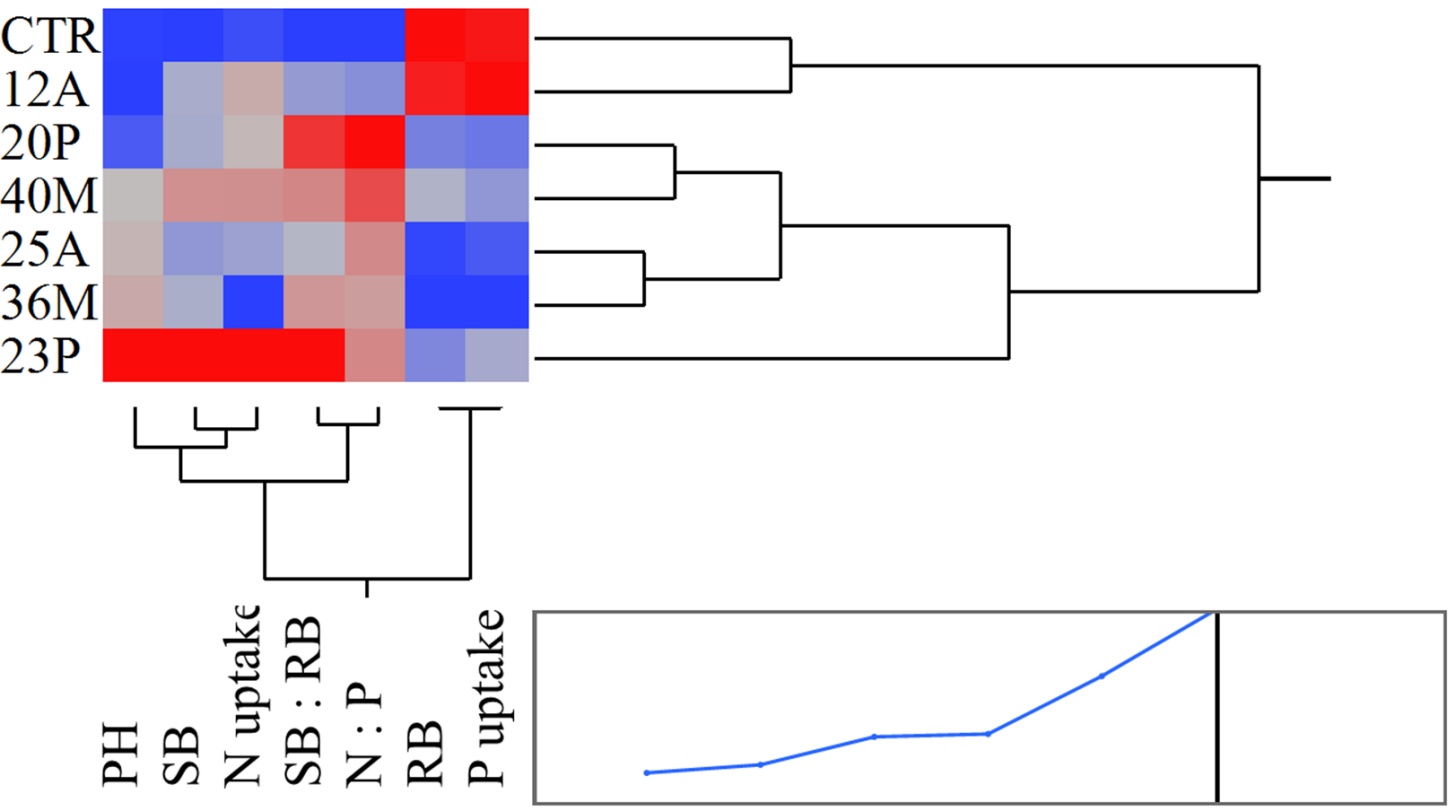
Heat map based on the hierarchical cluster analysis (Ward method) in relation to the response of durum wheat genotypes to different investigated wild PGPB strains.

**Figure 9:**
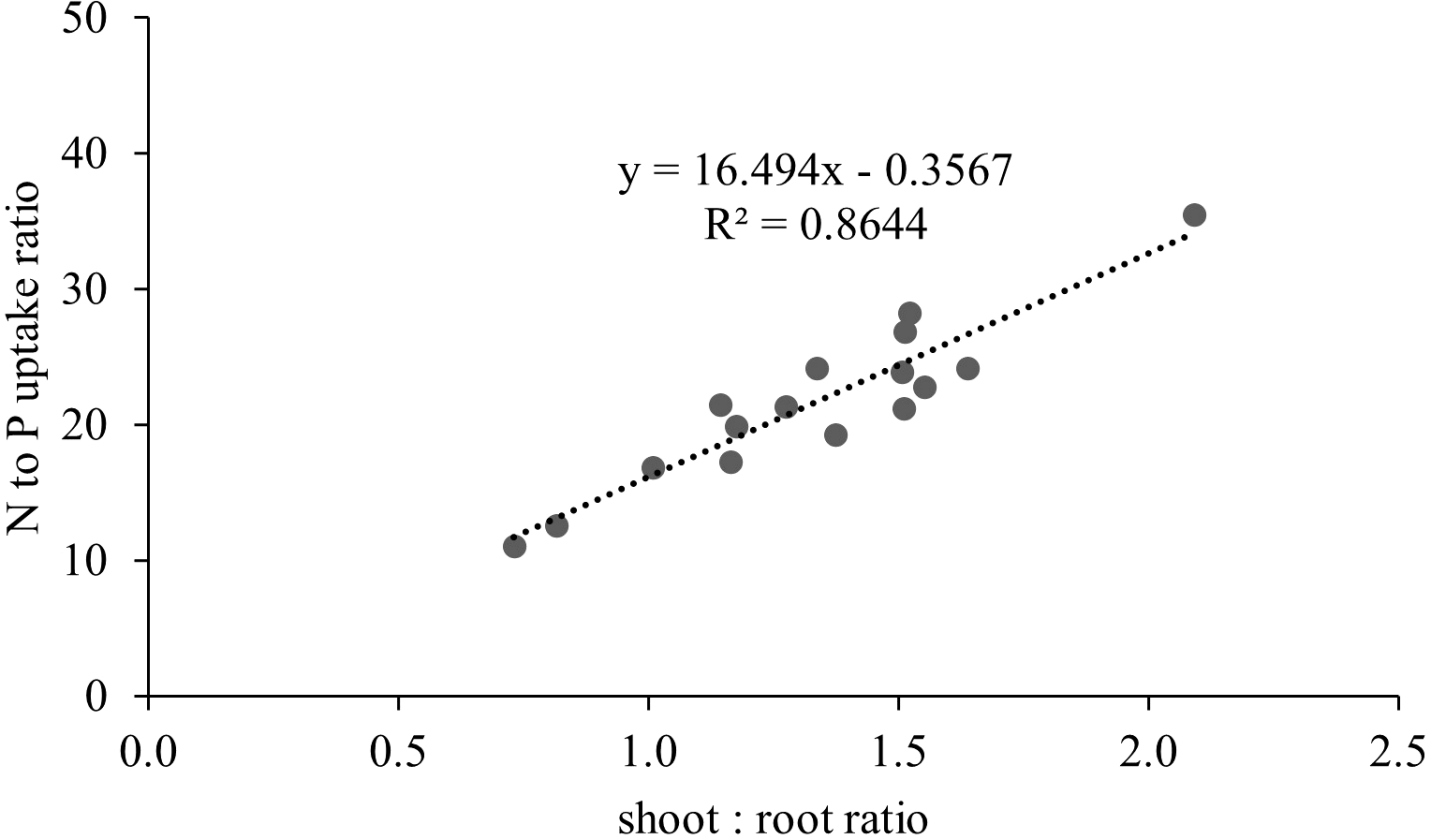
Relationship between shoot:root ratio and nitrogen to phosphorus uptake ratio in durum wheat inoculated with different wild PGPB strains under controlled conditions.

The inoculation of the investigated wild strains showed an impact on plant growth and mineral uptake of the durum wheat genotypes investigated under growth chamber conditions (**Table 5**). This resulted also in higher plant height (PH, **Figure 7**), that occurred from the initial stages with an increase of +20% *vs* CTR at 19 das (*p*<0.05; results of the hierarchical cluster analysis (**Figure 8**) indicate the 23P as the most effective PGPB strain, although also other strains (20P and 40M; 36M and 25A) clustered in a different class than the control. However, clustering showed the difference of 23P, which appeared to be the strongest effect in increasing plant biomass and N assimilation and along with shoot biomass (SB). On the other hand, root biomass (RB) showed a trend of reduction in the inoculated samples, with the exception of the strain 12A in Saragolla, which generally clustered in the same group of control (CTR). Due to the reduction of the root biomass, the ratio between shoot and root (shoot: root) was lower in the untreated control (CTR) with respect to the inoculated ones. As regards mineral assimilation, a trend of higher nitrogen (N) uptake (*p*<0.05) was observed in the treated durum wheat samples, with the exception of the 36M strain. Phosphorus (P) uptake, on the other hand, showed a trend of reduction in the inoculated samples, with the exception of the 12A strain, thus resulting in a general increase of the ratio between the N and P uptake (N:P) (*p*<0.05). Indeed, a strong relationship between the shoot: root and N:P ratios was found (**Figure 9**), as affected by PGPB inoculation.

**Table 5:** Effect of genotype and PGPB inoculation on shoot and root biomass, N and P accumulation and their ratio on two durum wheat genotypes grown under controlled conditions.

Source of variation	shoot	root	shoot: root	N uptake	P uptake	N:P
	g/m^2^	g/m^2^	ratio	mg/m^2^	mg/m^2^	ratio
Marco Aurelio	42.0	33.3	1.30	75.9	4.0	21.1
Saragolla	50.8	41.4	1.27	88.8	4.3	24.7
*p*	*	*	ns	*	ns	ns
CTR	39.6	45.3	0.87	75.3	5.5	14.3
12A	45.3	44.4	1.09	83.8	5.6	19.6
20P	45.2	34.9	1.33	83.0	3.7	27.7
23P	56.0	35.2	1.60	94.3	4.0	24.4
25A	44.2	32.6	1.34	80.5	3.5	24.4
36M	45.5	32.3	1.42	74.3	3.3	23.8
40M	48.9	36.8	1.34	85.6	3.9	26.0
LSD	7.0	8.1	0.37	11.4	0.8	5.7
*p*	*	*	*	*	*	*
interaction	ns	ns	ns	ns	ns	ns

CTR, untreated control; N, nitrogen uptake; P, phosphorus uptake; N:P, ratio between nitrogen and phosphorus uptake. LSD, least significant difference according to Tukey’s test as *post hoc*; ns, not significant; *significant difference at *p* < 0.05. Level of significance: ns, not significant.

As regards genotypic differences between the two durum wheat genotypes, Saragolla showed significantly higher shoot and root biomass (*p*<0.05), with no change in their ratio. Also, N uptake was significantly higher in Saragolla, while no significant genotypic changes in P uptake and N:P ratio. The interaction between durum wheat genotype and PGPB treatment was not significant for all the traits investigated.

Concerning microbiological analyses, the viable count of pseudomonads was higher in the soils inoculated with 23P and 20P (>1.5 log CFU/g), while spore forming bacteria had higher counts in the soils inoculated with *Bacillus* spp. (0.7-1.8 log CFU/g) (**Supplementary Table S1**); although a re-isolation of the strains was not done, the increase in pseudomonads and bacilli in some samples indirectly suggest the ability of the test strains to persist.

The last step was the agronomic validation of the 23P strain in the field trials. While for practical purposes and for the different kind of experiments, the application of PGPB was different and a foliar method was used, the results confirmed the effect of 23P strain on N, with consequent higher NUE (**Table 6**). The higher N uptake was also associated to an increase in the N:P ratio, consistent with the preliminary observation in the pot experiment. PUE, on the other hand, was not influenced by biofertilization. It is worth mentioning that the effect of PGPB on yield was significant in the soil characterized by lower fertility (S1). On the other hand, in S2 a higher GPC was observed with the PGPB inoculation. Lower PH was observed in the CTR only in S2.

**Table 6:** Effect of genotype and PGPB inoculation (23P strain) on different agronomic traits on two durum wheat genotypes grown in field trial in two different soils.

Soil	Source of variation	GY	GPC	PH	HI	N uptake	NUE	P uptake	PUE	N:P
		t/ha	%	cm	%	kg/ha	-	kg/ha	-	-
S1	Marco Aurelio	1.8	17.5	49	25.5	55	0.40	8.2	0.16	6.8
	Saragolla	2.1	16.0	46	29.3	59	0.46	6.2	0.12	9.5
	*p*	*	*	ns	*	ns	ns	*	*	*
	CTR	1.7	16.9	46	25.2	50	0.39	8.4	0.17	6.0
	23P	2.2	16.6	49	29.6	64	0.47	8.0	0.16	8.0
	*p*	*	ns	ns	*	*	*	ns	ns	*
	interaction	ns	ns	ns	ns	ns	ns	ns	ns	ns
S2	Marco Aurelio	3.0	16.7	87	25.2	88	0.68	8.1	0.16	10.9
	Saragolla	2.8	14.8	79	31.3	73	0.56	6.2	0.12	11.8
	*p*	ns	*	*	*	*	*	*	*	ns
	CTR	2.8	15.2	79	28.9	75	0.58	8.3	0.17	9.0
	23P	3.0	16.3	87	27.6	86	0.67	8.0	0.16	10.8
	*p*	ns	*	*	ns	*	*	ns	ns	*
	interaction	ns	ns	*	ns	ns	ns	ns	ns	ns

CTR, control; 23P, *Ps. migulae*; GY, grain yield; GPC, grain protein content; PH, plant height; HI, harvest index; N, nitrogen uptake; NUE, nitrogen use efficiency; P, phosphorus uptake; PUE, phosphorus use efficiency; N:P, ratio between nitrogen and phosphorus uptake. Different letters indicate significant differences according to Tukey’s test; ns, not significant; *significant difference at p < 0.05. Level of significance: ns, not significant.

As regards varietal differences, the higher productivity of Saragolla, that generally showed a higher HI and a lower PUE, was observed only under low fertility conditions (S1).

Concerning the microbiological data, in the combinations inoculated with 23P, pseudomonads were always 1.5–2 log CFU/g higher than in the other theses, while no differences were recorded for other groups (data not shown), thus indirectly confirming also in this case the ability of the strain to persist in soil.

## 4 Discussion

Management models for modern agriculture aim to promote the development of innovative technologies in the agricultural sector to improve food production and security, and to ensure sustainable adaptation to climate change (De Santis et al., 2024; Liu et al., 2022).

The efficacy of wild PGPB is reported in literature with positive effects on plant growth and nutritional assimilation (Ramakrishna et al., 2019; Rodríguez-Vázquez and Mesa-Marín, 2023); in addition, literature indicates the use of PGPB as a strategy to face abiotic and biotic stresses possibly increasing under climate change in the major crops, such as wheat (Brambilla et al., 2022; Carillo, 2025; Kumar et al., 2022). These conditions may be particularly relevant in the Mediterranean basin; thus, the selection of adapted PGPBs to improve wheat adaptability and resource use efficiency is then strategic (Cataldi et al., 2020; De Santis et al., 2024; Rossini et al., 2025; Yaghoubi Khanghahi et al., 2022; Zampieri et al., 2025).

This approach was investigated for some PGPB from durum wheat rhizosphere; they could be used as biofertilisers to improve nutrient use efficiency, yield and quality of durum wheat, especially in the Mediterranean area, where the environmental influence on crop performance is generally significant due to the considerable seasonal climatic variations (De Santis et al., 2024). Some authors have reported that PGPB have to face barriers and challenges, including the resistance to fungicides, the viability in soil also when subjected to a thermal stress, and growth under a wide variety of conditions (Hossain et al., 2023; Timmusk et al., 2017).

PGPB should be resistant to fungicides, as resistance to commonly used preparations is essential to ensure compatibility with conventional crop protection practices (Ahemad and Khan, 2012); moreover, it could assure the possibility of a combined treatment of seed with fungicides and PGPB for an effective management and application of both treatments in real conditions (De Andrade et al., 2023). Tolerance to fungicide is strongly strain-dependent and also relies upon the kind of compound/preparation and its concentration (Ahemad and Khan, 2012; Khan et al., 2020). In this research, 23P and 20P demonstrated the highest resistance to fungicides, thus suggesting the possibility of a combined application of these strains and fungicides, also at high concentrations.

In addition, soil persistence under temperature stress is critical for use in Mediterranean regions, where temperatures can exceed 40°C. The importance of PGPB effect at high-temperature stress has been extensively reviewed by Zhang et al. (2023); other authors (Bruno et al., 2020; Mishra et al., 2009; Selvakumar et al., 2008) reported the characterization of strains able to survive, persist and exert their effects also at high temperatures. The experiments showed that the strain 23P maintained a good persistence over time compared to other strains, thus suggesting a high potential for field application.

In addition, the ability of most strains to grow in a wide range of pH and temperature confirms their suitability and environmental adaptability and is in line with some literature evidence which stresses the adaptability of *Pseudomonas* and *Bacillus* genera to environmental fluctuations (Wang et al., 2023).

For the second step, the strains showed a variability in the plant response to inoculation. A general increase in ratio between above the ground biomass and root biomass and in the ratio between N and P uptake was observed, in accordance to literature (Caldara et al., 2024; Rossini et al., 2025). The promotion of N uptake in durum wheat due to microbial inoculation is associated to differential regulation of the nitrate transporter genes (Saia et al., 2015), together with an increase in above ground biomass. An element of novelty from this study is relative to the strong relationship between those ratios, in response to wild PGPB isolates. 23P emerged as the most efficient strain to improve biomass and mineral assimilation, with positive effects on NUE (Di Benedetto et al., 2019); the effectiveness of the microbial inoculation has been observed both in controlled conditions and in open field trial, indicating a general increase in NUE traits, for yield and protein content, depending on the level of soil fertility (Wu et al., 2022). The shoot: root ratio generally increases with the size for herbaceous plants (Poorter et al., 2012). Indeed, a decrease of this ratio is reported under P deficiency conditions (Liu, 2021). Further, the nitrogen to phosphorus (N:P) ratio is commonly assumed to reflect the relative availability of N and P in the soil in which the plant grows, especially stem and root (Garrish et al., 2010). This might contribute to explaining the relationship of the investigated mineral and biomass ratios traits, since the complexity of N and P interconnections (Krouk and Kiba, 2020), taking also into account that shoot biomass is driver for N uptake especially under low N supply (Kamiji et al., 2014). Also, root architectural traits might also explain the observed differences between the two durum wheat genotypes, which have been recently characterized for a different adaptability productivity (De Santis et al., 2024; Rajamanickam et al., 2024).

In conclusion, this study demonstrated that wild strains of PGPB from the rhizosphere of durum wheat exhibit variable but promising potential. The approach hereby proposed, based on a two-tiered methodology, was a suitable and a promising way also for future studies, and shows that the assessment of plant growth promoting traits is an important requisite, but not sufficient for an effective selection of promising PGPB. It is important to test the robustness of the strains also in relation to fungicides and their adaptability also to harsh conditions, simulated in this paper as an increasing temperature trends. The second step of the proposed tiered methodology is the evaluation of the most promising microorganisms under controlled conditions (growth chamber) and in field, also in relation to some agronomic variables. Focusing on the results, among the tested strains, 23P showed the highest technological robustness, with significant resistance to commercial fungicides, tolerance to thermal stress and strong persistence in soil. Its adaptability to a wide range of environmental conditions makes it a suitable candidate for practical application in the field; moreover, agronomic studies confirmed that 23P inoculation improved shoot biomass, nitrogen uptake and nitrogen use efficiency (NUE), especially under low fertility conditions, with consistent performance in both controlled and field environments. However, some points should be corroborated and validated in the future, that is multi-year persistence trials, the combination of 23P with reduced fertilization regimes, and the exploration of its performance in consortia with other beneficial microorganisms.

## Data Availability

The raw data supporting the conclusions of this article will be made available by the authors, without undue reservation.

## Supplementary material

Supplementary Table 1 Viable count (log CFU/g) of total aerobic bacteria (TVC), pseudomonads (PSE), enterobacteria (ENT), soil microorganisms at 22 °C (SOI), spore-forming (SPO) and actinobacteria (ACT) in the rhizosphere after 76 days. Experiments in growth chamber. Mean ± standard deviation.
