# Supplementary material for: A two-tiered methodology for the validation of promising plant growth promoting bacteria isolated from durum wheat rhizosphere
Source: Front Plant Sci. 2025 Nov 6;16:1707549. doi: 10.3389/fpls.2025.1707549 (PMC12632189; doi:10.3389/fpls.2025.1707549)
Supplement: Supplementary Table 1 — Viable count (log CFU/g) of total aerobic bacteria (TVC), pseudomonads (PSE), enterobacteria (ENT), soil microorganisms at 22 °C (SOI), spore-forming (SPO) and actinobacteria (ACT) in the rhizosphere after 76 days. Experiments in growth chamber. Mean ± standard deviation. [file Table1.docx]

Supplementary Material

**Supplementary Table S1.** Viable count (log CFU/g) of total aerobic bacteria (TVC), pseudomonads (PSE), soil microorganisms at 22°C (SOI), spore-forming (SPO) and actinobacteria (ACT) in the rhizosphere after 76 days. Experiments in growth chamber. Mean ± standard deviation.

The values of enterobacteria are not reported as they are below the detection limit.

| **MARCO AURELIO** | | | | | | |
| --- | --- | --- | --- | --- | --- | --- |
| **PGPB** | **TVC** | **SPO** | **PSE** | **ACT** | **SOI** | **pH** |
| 36M | 5.40 ± 0.09 | 5.10 ± 0.01 | 3.02 ± 0.06 | - | 5.31 ± 0.31 | 7.84 |
| 40M | 5.38 ± 0.22 | 5.42 ± 0.22 | 2.84 ± 0.22 | 6.48 ± 0.22 | 5.48 ± 0.22 | 7.82 |
| 6P | 5.18 ± 0.14 | 5.20 ± 0.10 | 3.65 ± 0.35 | 6.10 ± 0.30 | 5.16 ± 0.14 | 7.73 |
| 20P | 5.18 ± 0.31 | 4.18 ± 0.32 | 5.11 ± 0.21 | 6.18 ± 0.12 | 5.30 ± 0.10 | 7.82 |
| 23P | 5.04 ± 0.11 | 4.41 ± 0.01 | 5.04 ± 0.12 | 6.11 ± 0.11 | 5.50 ± 0.02 | 7.84 |
| 12A | 5.18 ± 0.22 | 5.18 ± 0.14 | 3.60 ± 0.00 | 6.10 ± 0.20 | 5.39 ± 0.31 | 7.79 |
| 25A | 5.46 ± 0.14 | 5.37 ± 0.23 | 3.54 ± 0.31 | 6.18 ± 0.14 | 5.26 ± 0.13 | 7.83 |
| CNT | 5.36 ± 0.33 | 4.41 ± 0.11 | 3.44 ± 0.02 | 6.24 ± 0.11 | 5.38 ± 0.22 | 7.85 |

| **SARAGOLLA** | | | | | | |
| --- | --- | --- | --- | --- | --- | --- |
| **PGPB** | **TVC** | **SPO** | **PSE** | **ACT** | **SOI** | **pH** |
| 36M | 5.22 ± 0.12 | 5.34 ± 0.11 | 3.28 ± 0.12 | 5.15 ± 0.10 | 5.36 ± 0.11 | 7.81 |
| 40M | 5.16 ± 0.11 | 5.26 ± 0.12 | 3.50 ± 0.30 | 5.15 ± 0.21 | 5.18 ± 0.22 | 7.85 |
| 6P | 5.15 ± 0.30 | 5.83 ± 0.12 | 3.26 ± 0.13 | 5.10 ± 0.22 | 5.38 ± 0.32 | 7.76 |
| 20P | 4.95 ± 0.31 | 4.15 ± 0.20 | 5.11 ± 0.21 | 5.18 ± 0.24 | 5.20 ± 0.10 | 7.87 |
| 23P | 4.84 ± 0.13 | 4.30 ± 0.10 | 5.15 ± 0.10 | 4.78 ± 0.12 | 4.81 ± 0.14 | 7.84 |
| 12A | 4.38 ± 0.02 | 5.20 ± 0.22 | 3.38 ± 0.12 | 5.16 ± 0.11 | 6.19 ± 0.11 | 7.77 |
| 25A | 4.38 ± 0.22 | 4.30 ± 0.30 | 3.38 ± 0.24 | 5.02 ± 0.23 | 5.37 ± 0.13 | 7.82 |
| CNT | 5.06 ± 0.44 | 3.04± 0.11 | 3.43 ± 0.12 | 5.22 ± 0.35 | 5.24 ± 0.12 | 7.85 |
